# Targeted Anticancer Immunotoxins and Cytotoxic Agents with Direct Killing Moieties

**DOI:** 10.1100/tsw.2006.162

**Published:** 2006-07-07

**Authors:** Koji Kawakami, Oumi Nakajima, Ryuichi Morishita, Ryozo Nagai

**Affiliations:** ^1^ Department of Pharmacoepidemiology, Graduate School of Medicine and Public Health, Kyoto University, Yoshida Konoecho, Sakyoku, Kyoto 606-8501, Japan; ^2^ Divison of Clinical Gene Therapy, Graduate School of Medicine, Osaka University, 2-2 Yamada-oka, Suita, Osaka 565-0871, Japan; ^3^ Department of Cardiovascular Medicine, Graduate School of Medicine, University of Tokyo, 7-3-1 Hongo, Bunkyoku, Tokyo 113-8655, Japan

**Keywords:** cancer, immunotoxin, cytotoxin, biological, clinical trial, receptor, antibody

## Abstract

Despite the progress of the bioinformatics approach to characterize cell-surface antigens and receptors on tumor cells, it remains difficult to generate novel cancer vaccines or neutralizing monoclonal antibody therapeutics. Among targeted cancer therapeutics, biologicals with targetable antibodies or ligands conjugated or fused to toxins or chemicals for direct cell-killing ability have been developed over the last 2 decades. These conjugated or fused chimeric proteins are termed immunotoxins or cytotoxic agents. Two agents, DAB_389_IL-2 (ONTAK^TM^) targeting the interleukin-2 receptor and CD33-calicheamicin (Mylotarg^®^), have been approved by the FDA for cutaneous T-cell lymphoma (CTCL) and relapsed acute myeloid leukemia (AML), respectively. Such targetable agents, including RFB4(dsFv)-PE38 (BL22), IL13-PE38QQR, and Tf-CRM107, are being tested in clinical trials. Several agents using unique technology such as a cleavable adapter or immunoliposomes with antibodies are also in the preclinical stage. This review summarizes the generation, mechanism, and development of these agents. In addition, possible future directions of this therapeutic approach are discussed.

## INTRODUCTION

A new form of cancer therapy has evolved, utilizing the knowledge of unique or overexpressed cell-surface antigens or receptors on tumor cells as the target. In particular, a variety of receptors for cellular growth factors and cytokines have been shown to be overexpressed on tumor cells, which may possibly serve as a target for cancer therapy. To target these receptors or antigens, antibodies or ligands conjugated or fused to natural toxins or chemicals, termed immunotoxins or cytotoxic agents, have been developed[1,2]. This review article summarizes the generation, mechanism, and clinical application of these molecules.

## PE-BASED IMMUNOTOXINS

To target cancer-specific antigens or receptors, immunotoxins can be produced in bacteria (such as *Escherichia coli*) as ligand-toxin fusion proteins, without requiring chemical conjugation of the antibody and toxin. *Pseudomonas* exotoxin (PE) is a 66-kDa pathogenic protein secreted by *P. aeruginosa* that irreversibly ADP-ribosylates the diphthamide residue of elongation factor 2 (EF2), using $NAD^+$ as a cofactor[2,3]. As a consequence, protein synthesis is inhibited and cell death occurs. PE is composed of three major domains. Domain I mediates binding to the α2 macroglobulin receptor. As shown in Figs. 1 and 2, this domain can be replaced by a ligand or variable domains of the antibody (Fv) to generate a cancer-specific immunotoxin. Domain II mediates translocation of domain III, the ADP-ribosylating domain, into the cytosol of target cells[4,5]. As shown in Fig. 2, on entry of PE into the cell, proteolytic cleavage occurs near Arg279, which generates a 28-kDa amino-terminal fragment and a 37-kDa carboxyl-terminal fragment[6]. The 37-kDa fragment contains the ADP-ribosylating activity of PE.

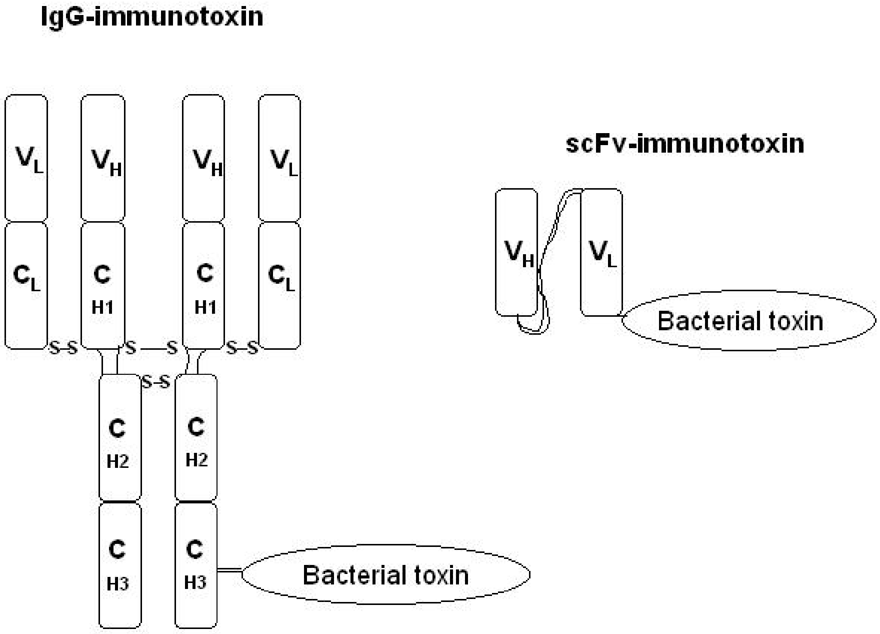

**FIGURE 1.** Schema for immunotoxins. The whole monoclonal antibody or scFv ($V_H$ and $V_L$ heterodimer) is conjugated with a truncated or mutated bacterial toxin, e.g., *Pseudomonas* exotoxin (PE), diphtheria toxin, ricin A chain toxin, or saporin toxin. In some cases, a cytokine ligand can be utilized instead of an antibody to generate a fusion protein.

Various efforts have been made to generate and develop PE-based immunotoxins[7,8]. These include anti-Tac(Fv)-PE38 (LMB-2), which targets CD25-positive leukemia, RFB4(dsFv)-PE38 (BL22) targeting CD22-positive B-cell malignancies, IL4(38-37)-PE38KDEL for the treatment of recurrent glioblastoma, and IL13-PE38QQR for the treatment of renal cell carcinoma and glioblastoma, which are currently undergoing clinical trials. Anti-Tac(Fv)-PE38 (LMB-2) contains the variable heavy domain ($V_H$) of the anti-tac MAb fused via the peptide linker $(G_4S)_3$ to the variable light domain ($V_L$), which in turn is fused to a 38-kDa truncated form of PE[9,10]. When administered to 35 patients with chemotherapy-resistant leukemia or lymphoma, anti-Tac(Fv)-PE38 treatment resulted in one complete remission (CR) and 7 partial responses (PR)[11,12]. RFB4(dsFv)-PE38 (BL22) is composed of the $V_L$ from the MAb RFB4 disulfide bonded to a fusion of $V_H$ with PE38[13]. When RFB4(dsFv)-PE38 was tested in 16 patients with hairy cell leukemia, the response rate was 81%, with 11 CRs and 2 PRs in 16 evaluable patients[14].

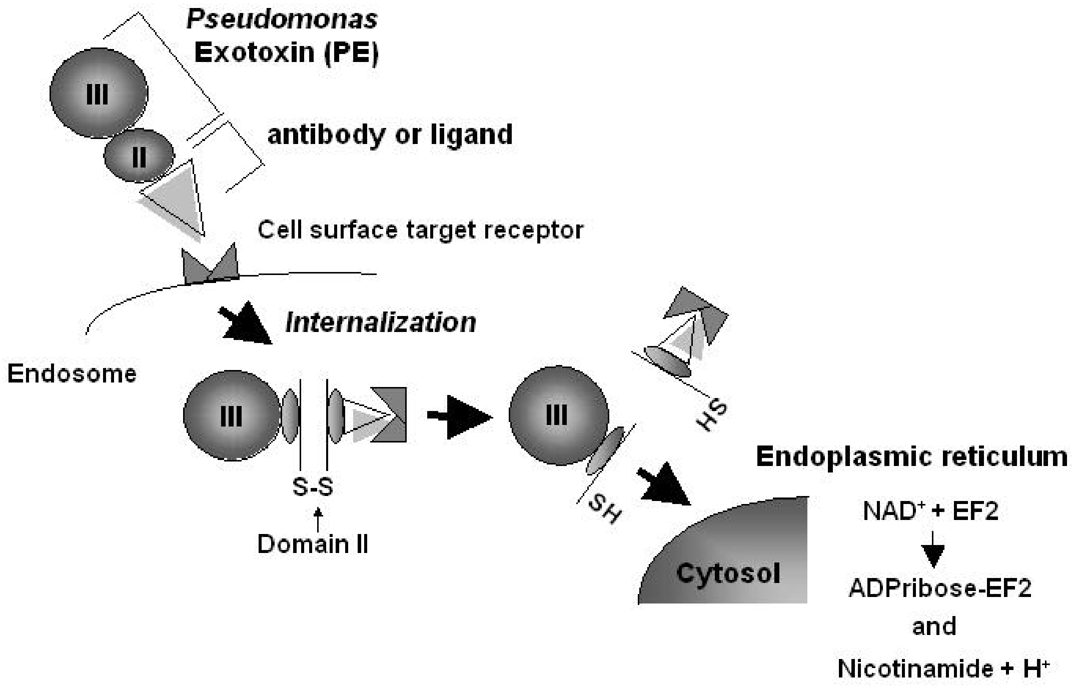

**FIGURE 2**. Cytotoxic mechanism of PE-based immunotoxin. The low-pH environment in the endosome causes unfolding of the immunotoxin at domain II. The carboxyl terminal fragment containing domain III, the ADP ribosylation domain, is transferred into the endoplasmic reticulum, then finally into the cytosol. It inhibits protein synthesis of targeted cells by irreversible ADP ribosylation of elongation factor (EF)-2, utilizing $NAD^+$ as a cofactor, resulting in cell death.

IL4(38-37)-PE38KDEL is a molecule containing amino acids 38–129 of interleukin (IL)-4, fused via a peptide linker to amino acids 1–37, which in turn are fused to amino acids 353–364 and 381–608 of PE, with KDEL (an endoplasmic retaining sequence)[15]. The receptor for IL-4 is expressed on various cancer cells, and IL4(38-37)-PE38KDEL has shown potent antitumor activity in a variety of cancer cells *in vitro* and *in vivo*[16]. In a phase I clinical trial, nine patients with recurrent brain tumor received IL4(38–37)-PE38KDEL by convection-enhanced delivery (CED)[17]. Tumor necrosis was observed in six patients, with one patient remaining disease-free for more than 18 months. An additional clinical trial is currently underway.

IL13-PE38QQR is a recombinant protein composed of human IL-13 and a mutated form of PE. This molecule has shown potent antitumor activity in animal models of glioblastoma multiforme (GBM)[18], AIDS-Kaposi's sarcoma[19], and head and neck cancer[20]. Based on preclinical results, three phase I/II clinical trials have been initiated in adults with recurrent GBM to determine the safety and tolerability of this agent. A multicenter phase III clinical trial in patients with recurrent GBM is currently ongoing in the U.S.

## DT-BASED IMMUNOTOXINS

Diphtheria toxin (DT) is a 535-amino-acid protein with three domains consisting of a catalytic domain also called the A fragment (amino acids 1–186) connected by a 14-amino-acid arginine-rich disulfide loop to a translocation domain followed by a flexible linker peptide (amino acids 382–390) and a β-sheet-rich cell-binding domain (amino acids 390–535)[21]. Similar to PE, the A fragment of DT catalyzes ADP-ribosylation of the diphthamide residue in domain IV of the elongation factor in the cytosol[22].

$DAB_{389}$IL-2 is a recombinant DNA-derived cytotoxic protein composed of the amino acid sequences for DT fragments A and B (Met1-The387)-His followed by the sequence for IL-2 (Ala1-Thr133)[23]. This molecule was developed for the treatment of chronic T cell lymphoma (CTCL) expressing CD25, and was the first FDA-approved immunotoxin[24]. DTAT (diphtheria toxin amino terminal fragment of urokinase-type plasminogen activator) is another DT-based immunotoxin targeting tumor vascular endothelium. DTAT has shown a potent antitumor activity in an animal model of malignant brain tumor[25].

Tf-CRM107 is another DT-based immunotoxin targeting malignant brain tumors[26]. This molecule is comprised of transferrin and a mutant DT, and CED administration of this immunotoxin has shown potent antitumor activity in a phase I clinical trial, in which 9 of 15 patients showed a >50% reduction in tumor volume[27].

Frankel and colleagues have generated and developed $DT_{388}$GMCSF for the treatment of acute myeloid leukemia (AML)[28]. This molecule is composed of amino acids 1–388 of DT, a histidine-methionine linker, and amino acids 1–124 of human granulocyte-macrophage colony stimulating factor (GMCSF)[29]. A phase I clinical trial has been completed in patients with AML, and 4 of 37 patients showed clinical remission of disease[30].

Another unique DT-based approach is VEGF121-DT385 and VEGF165-DT385 immunotoxins. Vascular endothelial growth factor (VEGF) is the most critical inducer of blood vessel formation. VEGF165 and VEGF121 are chemically conjugated to a truncated DT at residue 385 (DT385) containing a carboxyl terminus cystine residue. VEGF165 and VEGF121 were derivatized with the heterobifunctional agent *N*-succinimidyl-3-(2-pyridyldithio) propionate (SPDP) to introduce one to three activated thiol groups by modifying the ε-amino group of lysine residues[31]. *In viv*o animal studies have shown that these molecules are able to inhibit angiogenesis and tumor growth[31,32].

## RICIN-BASED IMMUNOTOXINS

Ricin is a plant lectin from the seeds of the castor plant *Ricinus communis* that belongs to the class of ribosome-inhibiting proteins (RIPs). The A-chain (30 kDa), conferring RNA N-glycosidase activity to cleave a specific adenine base from ribosomal RNA, is connected through a disulfide linkage with the B-chain (32 kDa). After being internalized into the cell, the interchain disulfide-bond is reduced before the catalytic A-chain domain is delivered to the ribosomes where it inactivates the 60S ribosomal subunit required for the binding of elongation factor-2 during protein synthesis. The enzymatic A-chain hydrolyzes an adenine-ribose linkage within the 28S rRNA, thereby inhibiting protein synthesis[33,34].

A variety of ricin-based immunotoxins against non-Hodgkin lymphoma (NHL) expressing CD5, CD19, and CD22 have been tested in phase I and II clinical trials, with limited efficacy[35]. A major adverse event in the toxicity profile was vascular leak syndrome (VLS). Messmann et al. reported the use of a cocktail of two ricin A-chain immunotoxins (RFB4-SMPT-dgA and IgG-HD37-dgA) in NHL patients; however, the clinical outcome was moderate as only 2 of 22 patients showed a partial response (PR)[36].

Ricin A-chain immunotoxins against CD25 (RFT5.dgA) or CD30 (Ki-4.dgA)-positive Hodgkin’s lymphoma have also been exploited in preclinical and clinical studies. PR rate in clinical studies was 2/15 in the CD25 trial and 1/17 in the CD30 trial[37,38].

## SAPORIN-BASED IMMUNOTOXINS

Saporin is a single chain RIP that has no lectin-like binding activity, originating from the seeds of the soapwort plant (*Saponaria officinalis*)[39,40]. Similar to the ricin A-chain, saporin acts as an N-glycosidase catalytically cleaving 28S ribosomal RNA at A-4324, thus irreversibly inactivating cellular ribosome capability[41].

Several saporin-based immunotoxins have been generated and developed; however, their clinical experience is very limited[42]. Anti-CD30-saporin (BER-H2/SO6) has been used in advanced refractory Hodgkin's lymphoma. Although no major toxicity was observed, the response rate was 40% of 12 patients and was transient[43].

Fuchs and colleagues have developed a unique saporin-based toxin, containing saporin toxin linked to epidermal growth factor (EGF) via an adapter composed of a cytosolic cleavable peptide (CCP), membrane transfer peptide (MTP), and endosomal cleavable peptide (ECP)[44]. The molecule with the adapter showed a reduced half-life without compromising the cytotoxic activity to cancer cells *in vitro*, having the advantage of less immunogenicity and nonspecific toxicity.

## ANTIBODY-CONJUGATE CYTOTOXIC AGENTS

In contrast to utilizing natural toxins, several attempts have been made to develop the tumor-specific antibody chemically conjugated to cytotoxic drug that is being used for the chemotherapy of the cancer[45]. Among them, anti-CD33-calicheamicin immunoconjugate (gemtuzumab ozogamicin, Mylotarg®) for the treatment of AML was granted marketing approval by the FDA on May 17, 2000 under the Accelerated Approval regulations[46]. Calicheamicin is a highly active DNA-cleaving agent that introduces sequence-selective double-stranded cuts[47]. As shown in Fig. 3, the hydrazide derivative of calicheamicin, NAc-γ-calicheamicin, was linked to a humanized form ($IgG_4$) of the anti-CD33 antibody, anti-hP67.6, to generate gemtuzumab ozogamicin[48]. It has been shown that this conjugated molecule has extremely selective activity against leukemic cells[49]. In preapproval trials, patients with relapsed or refractory CD33-positive AML ($n$ = 277) were treated with gemtuzumab ozogamicin[50,51]. The overall response rate was 30%. Grade 3/4 liver toxicity (hepatic veno-occlusive disease) was observed in 3–5% of patients. It is noteworthy that no humoral immune response to the anti-hP67.6 antibody was detected in the trials[52]. In addition, another calicheamicin conjugate, CMC-544, is linked to humanized anti-CD22 monoclonal antibody, which is being evaluated in B-cell non-Hodgkin's lymphoma patients. It has been reported that a combination of CMC-544 and Rituximab shows superior antitumor activity in preclinical studies in animal models of B-cell lymphoma[53]. Phase I clinical trials to assess safety and tolerability of this molecule are currently ongoing.

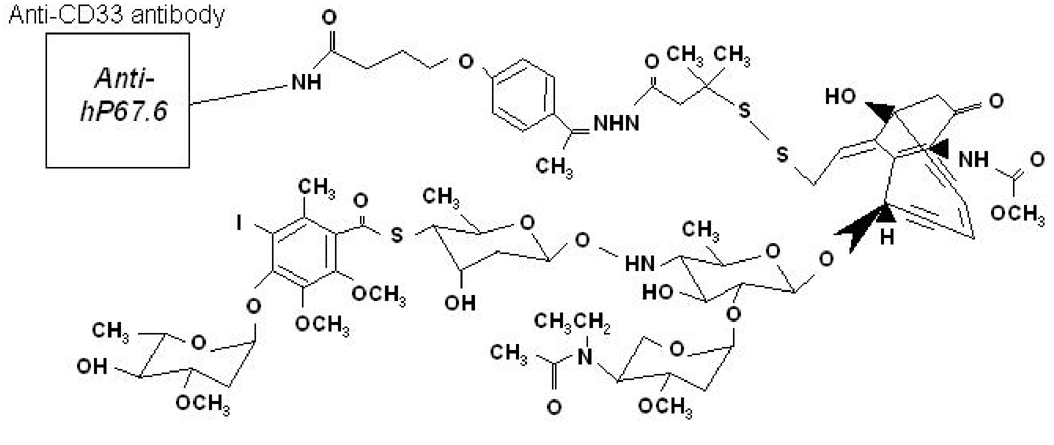

**FIGURE 3.** Gemtuzumab ozogamicin (Mylotarg®). NAc-γ-calicheamicin is linked to the humanized form ($IgG_4$) of anti-CD33 monoclonal antibody, anti-hP67.6.

Another chemical-conjugate cytotoxic agent falling into this category is BR96-doxorubicin (SGN-15). BR96-doxorubicin is a chimeric protein composed of the monoclonal antibody BR96, which targets tumor cells expressing a LewisY ($Le^Y$)-related antigen and the chemotherapeutic drug doxorubicin[54]. Because an acid-labile linker is utilized to conjugate antibody and doxorubicin, once BR96-doxorubicin enters an acidic environment such as lysosomes and endosomes, doxorubicin is cleaved by hydrolysis and is able to exert its cytotoxic effects locally. This molecule is cytotoxic to various cancer cell lines[54] and shows a strong antitumor effect in combination with paclitaxel[55]. Results from phase I trials with BR96-doxorubicin as a single agent or BR96-doxorubicin in combination with docetaxel demonstrated no major side effects in patients with breast or colorectal cancer[56,57]. Developing of BR96-doxorubicin, however, was discontinued in the year 2005.

To date, four maytansinoid-based cytotoxic agents are being developed. Maytansinoid 1 and 4 (termed DM1 or DM4) are microtubule-depolymerizing compounds. DM1 or DM4 are conjugated to various antibody and the resulting cytotoxic agents, huN901-DM1 (BB-10901), MLN2704, huC242-DM4, and huMy9-6-DM4 (AVE9633) are currently in clinical trials[58,59,60,61].

HuN901-DM1, a compound comprised of a CD56-targeted humanized N901 antibody conjugated to DM1, is being developed for the treatment of CD56-expressing cancers including small-cell lung cancer[58]. This agent is being tested in phase I/II clinical trial for the treatment of small-cell lung cancer. Another DM1-based drug, MLN2704, is DM1 linked to a deimmunized monoclonal antibody that is specific for prostate-specific membrane antigen (PSMA). PSMA is a transmembrane receptor expressing on prostatic epithelium and prostate cancer cells, and it has been reported that its expression levels are positively collerated with the progression of malignancy[59]. This agent is being tested in a phase I/II clinical trial for the treatment of prostate cancer. HuC242-DM4 is a conjugation of DM4 to the humanized anti-CanAg monoclonal antibody. The CanAg antigens are expressed on colorectal, pancreatic, and certain nonsmall-cell lung cancers. This drug is currently being tested in a phase I clinical trial in patients with advanced solid tumors expressing CanAg (e.g., colon cancer and pancreatic cancer)[60].

HuMy9-6-DM4 (AVE9633) is a conjugation of DM4 to humanized anti-CD33 monoclonal antibody targeting AML. Phase I clinical trial to assess safety and tolerability of this molecule are being initiated in patients with AML.

There are several other chemical compound-based targeted cytotoxic agents in preclinical stages. Chemical compounds such as DNA alkylating agent (CC-1065)[61], a second-generation taxane[62], monometyl auristatin E[63], and geldanamycine[64] are conjugated to several monoclonal antibodies by difulfide linkers specifically designed to release drug into the targeting cancer cells.

## FUTURE DIRECTIONS OF IMMUNOTOXINS

The experience with immunotoxins indicates that immunogenicity of toxin in humans, expression levels in cancer cells, and specificity of the molecule to cancer cells are the major issues to be resolved for the future development of this category of biologicals.

To increase the opportunity for the immunotoxin to bind to the target tumor cells, Vallera et al. generated a bispecific DT-based immunotoxin that contains sFv of CD19 and CD22 (DT2219)[65]. It has been shown that this molecule is cytotoxic to $CD19^+CD22^+$ cells such as B-cell leukemia and lymphoma.

A urokinase-activated recombinant diphtheria toxin targeting the GMCSF receptor (DTU2GMCSF) is an innovation to give an immunotoxin the capability of dual specificity to AML cells[66]. Since a tumor-selective urokinase plasminogen activator (uPA)-uPA receptor system is overexpressed in various tumor cells including leukemias[67], a furin cleavage region ($_{163}RVRRSV_{170}$) in $DT_{388}$GMCSF was converted to a uPA cleavage sequence ($_{163}GSGRSA_{170}$). It was shown that the resulting molecule, DTU2GMCSF, was specific to AML cell lines with less toxicity to normal cells expressing the uPA receptor or GMCSF receptor alone.

To decrease immunogenicity and increase the plasma half-life and stability of the immunotoxin, Tsutsumi et al. introduced a peptide connector between the Fv and the toxin portion of Tac(Fv)-PE38, followed by site-specific conjugation with 5 or 20 kDa of polyethylene glycol (PEG)[68]. This modification rendered a five- to eightfold increase in the plasma half-life in mice, and a three- to fourfold increase in antitumor activity against ATac-4 tumor in mice.

In conclusion, immunotoxins are tumor-directed biologicals with a targetable moiety that possess the possibility to cure solid and hematologic malignancies. Although currently only two drugs have been approved by the FDA, a variety of potent agents are in clinical trials. New discoveries and further knowledge of tumor surface antigens and modifications, e.g., dual-specificity and PEGylation, will improve the application of immunotoxins to cancer.
